# Genotype × environment interaction of lowland bread wheat varieties for irrigation in different areas of Oromia

**DOI:** 10.1002/pei3.10097

**Published:** 2023-01-02

**Authors:** Tilahun Bayissa, Girma Mengistu, Geleta Gerema, Urgaya Balcha, Hailu Feyisa, Aliyi Kedir, Zeleke Legese, Desu Asegid, Tesfaye Leta, Tafa Jobe

**Affiliations:** ^1^ Sinana Agricultural Research Center Bale Robe Ethiopia; ^2^ Oromia Agricultural Research Institute Addis Ababa Ethiopia; ^3^ Bako Agricultural Research Center Bako Ethiopia; ^4^ Adami Tulu Agricultural Research Center Batu Ethiopia; ^5^ Bore Agricultural Research Center Bore Ethiopia; ^6^ Fedis Agricultural Research Center Harer Ethiopia; ^7^ Mechara Agricultural Research Center Mechara Ethiopia

**Keywords:** AMMI, IPCA, irrigated wheat, lowland, stable variety

## Abstract

Ethiopia is the leading wheat producer in Sub‐Saharan Africa, and the productivity has increased in the last few years. There is also a potential for irrigated wheat production in the lowlands, even though its cultivation is at infant stage. The experiment was conducted in the Oromia region at nine locations in 2021 with irrigation. The study aimed to select high yielding and stable bread wheat variety/ies for lowland areas. Twelve released bread wheat varieties were tested using randomized complete block design with two replications. Environment had the largest effect, 76.5% of total variability, while genotypes 5.0% and GE interaction 18.5% explained total sum of squares. The average grain yield of varieties across locations ranged from the lowest 1.40 t ha^−1^ at Girja to the highest 6.55 t ha^−1^ at Daro Labu, with a grand mean of 3.14 t ha^−1^. The result showed that varieties released for irrigated areas, Fentale 1, Ardi, and Fentale 2, were ranked the top three based on overall environment mean grain yield. The first and second principal component account 45.5% and 24.7% of the genotype by environment interaction (G × E), respectively, explained 70.2% of the total variation. Daro Lebu and Bedeno were the most productive environment, while Girja was the least productive of irrigated bread wheat for lowlands of the Oromia region. Genotype Selection Index (GSI) showed that varieties Fentale 2, Fentale 1, Pavon 76, and ETBW9578 are stable and high yielding. Based on AMMI and GGE biplot analysis, Girja indicated the most discriminating area and Sewena as representative environment for selecting wide adaptable irrigated lowland varieties. The results of the present study indicated that Fentale 2 and Fentale 1 showed better yield stability across all test environments, therefore, these bread wheat varieties are recommended for wide cultivation in irrigated areas of the Oromia region.

## Graphical abstract

Twelve bread wheat varieties were evaluated in nine locations. The highest grain yield of 6.55 t ha^−1^ was obtained during variety testing. Fentale 2 and Fentale 1 varieties gave better yield stability across all locations were recommended.

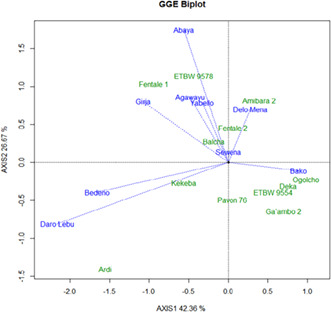

## 1 INTRODUCTION

Wheat (*Triticum aestivum* L.) is one of the most important cereal crop produced worldwide. It is one of the strategic crop in Ethiopia, because of its role in food security, import substitution, and supply of raw material for the agro‐processing industry. Ethiopia is a leading wheat producer in Sub‐Saharan Africa with total production of 4.6 million tons (CSA, 2018; FAO, 2018). Although the productivity of wheat increased in the last few years in the country, however, it is still very low as compared to other wheat‐producing countries in the world. The national average wheat productivity is estimated to be 3.05 t ha^−1^ (CSA, 2020), which is below the world average of 3.5 t ha^−1^. Despite the recent production increment, Ethiopia is still importing about 1.6 million tons of wheat which is estimated about 25% in deficit to fulfill domestic wheat demand (USDA, 2018).

Ethiopia is one of the few African countries endowed with relatively abundant water resources, favorable climate, and potentially huge irrigable land. Although there is a large coverage for irrigated areas, the actual achievement in many irrigated areas of the country is substantially less than the potential (MoWR, 2005). Several bread wheat varieties have been released for rain‐fed production targeted for different midland and highland agro‐ecologies. However, limited number of high yielding and stable released wheat varieties accessed for irrigated lowland area of the region (Tadesse & Assefa, 2018).

In most of the plant breeding programs, GE interaction effects are of special interest for identifying the most stable genotypes for mega‐environments and adaptation for specific targets. Various methods are used for stability analysis based on different stability concepts and can be classified accordingly (Flores et al., 1998). Information regarding crop stability is applicable for the selection of genotypes with constant yield across environments. Many of researchers have been reported to depict the responses of genotypes to the different condition of environments for simultaneous selection of yield and stability. These techniques are using statistical parameters to estimate the stability of genotypes to variation in environments. Linear regression approach is used widely used to identify high yielding and stable genotypes (Alberts, 2004).

The additive main effect and multiplicative interaction (AMMI) method is an approach for evaluation of genotypes stability under different environments. The AMMI method merges principal components analysis and analysis of variance into an integrated approach and can be used to analysis of the multi‐location experiments (Zobel et al., 1988). The AMMI analysis is effective because it provides agronomically meaningful interpretation of data (Gauch, 1992). The AMMI model is utilized for three main purposes (Crossa et al., 1990; Gauch, 1988): (i) to suitable in the initial statistical analyses of yield experiments, (ii) to summarize the relationships between genotypes and environments (GE) and (iii) it is applicable for understanding complex genotypes × environment interaction effects. AMMI analysis has been applied extensively with great success to interpret genotype × environment interaction in wheat (Mohammadi et al., 2013; Petrovic et al., 2010). A wider adapted genotype performs consistently over a wider range of environment. Therefore, the objective of this study was to identify stable, high yielding and best adapted bread wheat varieties for lowland irrigated areas of the Oromia region.

## 2 MATERIALS AND METHODS

### 2.1 Experimental design and methods

**FIGURE 1:**
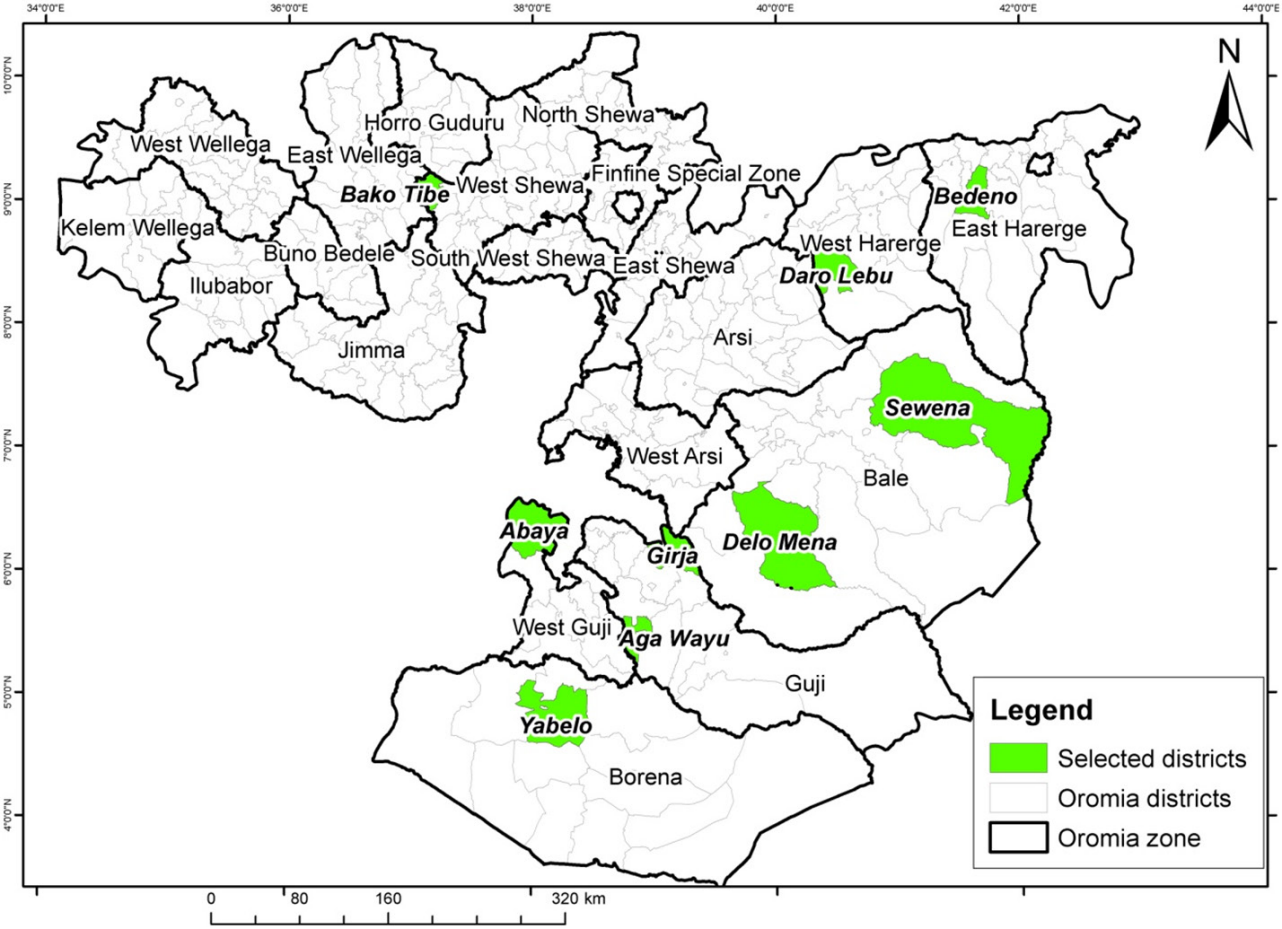
Map of Oromia showing selected lowland districts for irrigated wheat production

The experiment was conducted in Oromia region during 2021 off‐season using irrigation at Bako, Abaya, Girja, Agawayu, Bedeno, Daro Labu, Delo Mena, Sewena, and Yabelo (Figure 1). A total of 12 released bread wheat varieties for rain‐fed and irrigation (Table 1) were tested using a randomized complete block design with two replications. A plot of 10 rows with 0.3 meter row spacing and with 0.6 meter between ridges and 5 meter row length were used and the eight middle rows were used for data collection. About 120 kg ha^−1^ seed rate and fertilizer were applied at the rate of 100 kg ha^−1^ NPS and 150 kg ha^−1^ urea based on previous recommendations in the irrigated areas of Oromia. Urea was applied on split basis; 1/3 at planting and the remaining 2/3 at tillering stage of the crop. Other management practices were performed as per previous recommendations.

**TABLE 1:** List of bread wheat varieties, pedigree, releasing research center, and year of release

S/N	Variety name	Pedigree	Released by	Year of release	Released for
1	Deka	ATTILA/3*BCN*2//BAV92/3/KIRITATI/WBL1/4/DANPHE	KARC	2018	Rain‐fed areas
2	Balcha	CROC_1/AE.SQUARROSA (213)//PGO/10/ ATTILA*2/9/KT/BAGE//FN/U/3/ BZA/4/TRM/5/ALDAN/6/SERI/7/VEE#10/8/OPTA	KARC	2019	Rain‐fed areas
3	ETBW 9578/Dursa	NAVJ07/SHORTENED SR26 TRANSLOCATION/3/ATTILA/BAV92//PASTOR	KARC	2020	Rain‐fed areas
4	ETBW 9554/Boru	AUAL/MUTUS/6/CNO79//PF70354/MUS/3/PASTOR/4/BAV92*2/5/FH6‐1‐7/7/CNO79//PF70354/MUS/3/PASTOR/4/BAV92*2/5/FH6‐1‐7	KARC	2020	Rain‐fed areas
5	Ardi	GLADIUS/2*BAVIS	WARC	2019	Irrigated areas
6	Ga'ambo‐2	HEILO//MILAN/MUNIA/3/KIRITATI/2*TRCH	WARC	2019	Irrigated areas
7	Fentale 1	MOONTIJ‐3(FERROUG‐2/FOW‐2)	WARC	2015	Irrigated areas
8	Fentale 2	QAFZAH‐2/FERRIUG‐2	WARC	2017	Irrigated areas
9	Amibara 2	ETBW 5963	WARC	2017	Irrigated areas
10	Kekeba	Picaflor #1	KARC	2010	Rain‐fed areas
11	Pavon 76	‐	KARC	1982	Rain‐fed areas
12	Ogolcho	ETBW5520	KARC	2012	Rain‐fed areas

Abbreviations: KARC, Kulumsa Agricultural Research Center; WARC, Werer Agricultural Research Center.

### 2.2 Statistical analysis

Mean grain yield data of the experiment were statistically treated by additive main effect and multiplicative interaction (AMMI) model analysis. This analysis consists of the sequential fitting of a model of analysis of experiments, initially by ANOVA (additive fitting of the main effects) and then by analysis of principal components (multiplicative fitting of the effects of interaction). The model AMMI equation is:

$$Y_{\mathrm{ij}}=\mu +g_{i}+e_{j}+\sum n=1^{h}\lambda_{n}\alpha_{\mathrm{ni}}.Y_{\mathrm{nj}}+R_{\mathrm{ij}}$$

where Yij is the yield of the ith genotype in the jth environment; μ is the grand mean; gi and ej are the genotype and environment deviations from the grand mean, respectively; λ_n_ is the square root of the eigen value of the principal component analysis (PCA) axis, α_ni_ and Y_nj_ are the principal component scores for the PCA axis n of the ith genotype and jth environment, respectively, and; R_ij_ is the residual. The analysis was done using R software (R for windows) version 4.1.

### 2.3 AMMI stability value (ASV)

The ASV is the distance from the coordinate point to the origin in a two‐dimensional IPCA1 score against IPCA2 scores in the AMMI model (Purchase et al., 2000). Because of the IPCA1 score contributes more to the GE interaction sum of square, a weighted value is needed. This weight is calculated for each genotype and environment according to the relative contribution of IPCA1 to IPCA2 to the interaction SS as follows,

$$\mathrm{ASV}=\sqrt{{\left\lfloor \frac{\text{SSIPCA}1}{\text{SSIPCA}2}\left(\text{IPCA}1\text{Score}\right)\right\rfloor }^{2}+{\left[\text{IPCA}2\right]}^{2}}$$

where SSIPCA1/SSIPCA2 is the weight given to the IPCA1 value by dividing the IPCA1 sum squares by the IPCA2 sum of squares. The larger the IPCA score, either negative or positive, the more specifically adapted a genotype is to certain environments. Smaller IPCA score indicate a more stable genotype across environment.

### 2.4 Genotype selection index (GSI)

Based on the rank of mean grain yield of genotypes (rYSI) across environments and rank of AMMI Stability Value (rASV) a genotype selection index GSI was calculated for each genotype which incorporates both mean grain yield and stability index in a single criterion as suggested by Bose et al. (2014) and Bavandpori et al. (2015):

GSI=rASV+rYSI

## 3 RESULTS AND DISCUSSION

### 3.1 Varietal evaluation

Homogeneity of variance tests indicated homogenous error variance for grain yield in the nine environments allowed for a combined analysis across environments. The combined analysis of variance (ANOVA) for grain yield showed highly significant differences (*p* < 0.01). Average grain yield of varieties across location ranged from the lowest 1.40 t ha^−1^ at Girja to the highest 6.55 t ha^−1^ at Daro labu, with a grand mean of 3.14 t ha^−1^ (Table 2). The observed varieties mean grain yield across environments ranged from the lowest 1.95 t ha^−1^ for Girja to 4.81 t ha^−1^ for Daro labu (Table 2). Mean comparison for the tested varieties indicated that maximum grain yield was obtained from Fentale 1 (3.62 t ha^−1^) followed by ETBW 9578 (3.37 t ha^−1^), Ardi (3.28 t ha^−1^) and Fentale 2 (3.24 t ha^−1^) whereas the least mean grain yield was obtained from Deka (2.79 t ha^−1^). The result showed that varieties released for irrigated area Fentale 1, Ardi, and Fentale 2, were ranked the first top three based on overall environment mean grain yield. However, varieties released for rain‐fed Deka was least performance among the tested genotypes (Table 2).

**TABLE 2:** Mean performance of 12 bread wheat genotypes in 9 environments

Variety	Mean grain yield (t ha^−1^) in tested environments									Combined mean yield (t ha^−1^)
	Abaya	Agawayu	Bako	Bedeno	Daro Lebu	Delo Mena	Girja	Sewena	Yabello	
Amibara 2	4.05	2.45	3.05	3.55	4.45	2.90	1.70	2.60	4.15	3.21
Ardi	2.65	2.40	2.30	5.40	6.55	1.95	1.85	2.55	3.85	3.28
Balcha	3.55	2.20	1.85	3.95	4.80	2.35	1.60	2.75	4.75	3.09
Deka	2.90	2.05	2.45	3.50	4.05	2.40	1.50	2.60	3.65	2.79
ETBW 9554	2.75	2.35	2.75	3.65	4.35	2.35	1.55	2.60	3.80	2.91
ETBW 9578	3.60	3.15	2.35	3.75	4.75	2.60	3.00	2.45	4.70	3.37
Fentale 2	3.50	3.10	2.80	4.00	4.55	2.55	1.80	2.90	3.95	3.24
Fentale 1	4.20	3.35	2.80	4.80	5.20	2.90	2.60	2.80	3.95	3.62
Ga'ambo‐2	2.25	2.65	3.15	3.50	4.45	2.55	1.65	2.50	3.60	2.92
Kekeba	3.15	2.65	2.70	4.00	5.40	1.95	2.70	2.45	3.45	3.16
Ogolcho	2.90	2.55	3.80	3.75	3.95	2.40	1.40	2.65	3.65	3.01
Pavon 76	2.75	2.10	3.00	3.70	5.20	2.65	2.05	2.65	3.90	3.11
Mean	3.19	2.58	2.75	3.96	4.81	2.46	1.95	2.63	3.95	3.14
LSD 0.05	0.88	0.43	0.97	0.18	0.85	0.42	0.71	0.88	1.60	0.27
CV (%)	15.32	9.18	19.68	2.54	9.82	9.52	20.24	18.76	22.56	15.26

Underlined numbers indicate the highest mean grain yield (t ha^−1^) at tested environments and the highest combined mean yield (t ha^−1^).

### 3.2 AMMI analysis

The combined analysis of variance indicated that the main effects of random environments and fix genotypes were significant for grain yield that exhibited the presence of variability in genotypes and diversity of growing conditions at different environments. The combined analysis of variance was conducted to determine the effects of environment (location), genotype and their interactions on grain yield of lowland irrigated bread wheat varieties (Table 3). The main effects of environment (E), genotype (G), and GE interaction were highly significant at *p* < 0.01. Environment had the largest effect, explaining 76.5% of total variability, while genotypes and GE interaction explained 5.0 and 18.5% of total sum of squares, respectively (Table 3). A large contribution of the environment indicated that environments were diverse, with large differences among environmental means causing most of the variation in grain yield and higher differential in discriminating the performance of the genotype. The same result was reported by Farshadfar (2008), Jacobsz et al. (2015), and Tadesse et al. (2017). The G × E interaction effect was almost three times higher than the genotypic effect. This may indicate the existence of a considerable amount of deferential response among the genotypes to changes in growing environments and the differential discriminating ability of the test environments.

**TABLE 3:** ANOVA for grain yield of bread wheat genotypes for the AMMI model

Source	Df	SS	MSS	Explained SS%
Genotypes	12	10.54	0.88**	5.0
Environments	8	161.29	20.16**	76.5
Replication (Environment)	1	0.07	0.07	
Interactions	96	38.93	0.41**	18.5
IPCA 1	19	17.37	0.91**	45.5
IPCA 2	17	10.40	0.61**	24.7
IPCA 3	15	6.23	0.42*	13.1
Residuals	90	21.44	0.24	

Abbreviations: df, degree freedom; SS, sum of square; MSS, mean sum of square, SS%, percentage of sum of square, IPCA 1, 2, and 3 = first, second, and third principal component.

*, ** Significant at 5% and 1% level of significance, respectively.

The combined analysis of variance and AMMI analysis is shown in Table 3. The AMMI model analysis of variance for grain yield showed highly significant differences (*p* < 0.01) for genotypes, environments, and genotype‐by‐environment interactions. The first two principal component axis of G × E was also highly significant (*p* < 0.01). The first and second principal component analyses accounted for 45.5% and 24.7% of the G × E, respectively, together explained 70.2% of the total variation (Table 3). This was in agreement with Mattos et al. (2013); Regis et al. (2018) suggested that G × E pattern is collected in the first two principal component analyses. Similarly, previous studies also suggested the importance of capturing most of the G × E sum squares in the first two principal component axis to attain accurate information (Crossa et al., 1990; Purchase et al., 2000).

**FIGURE 2:**
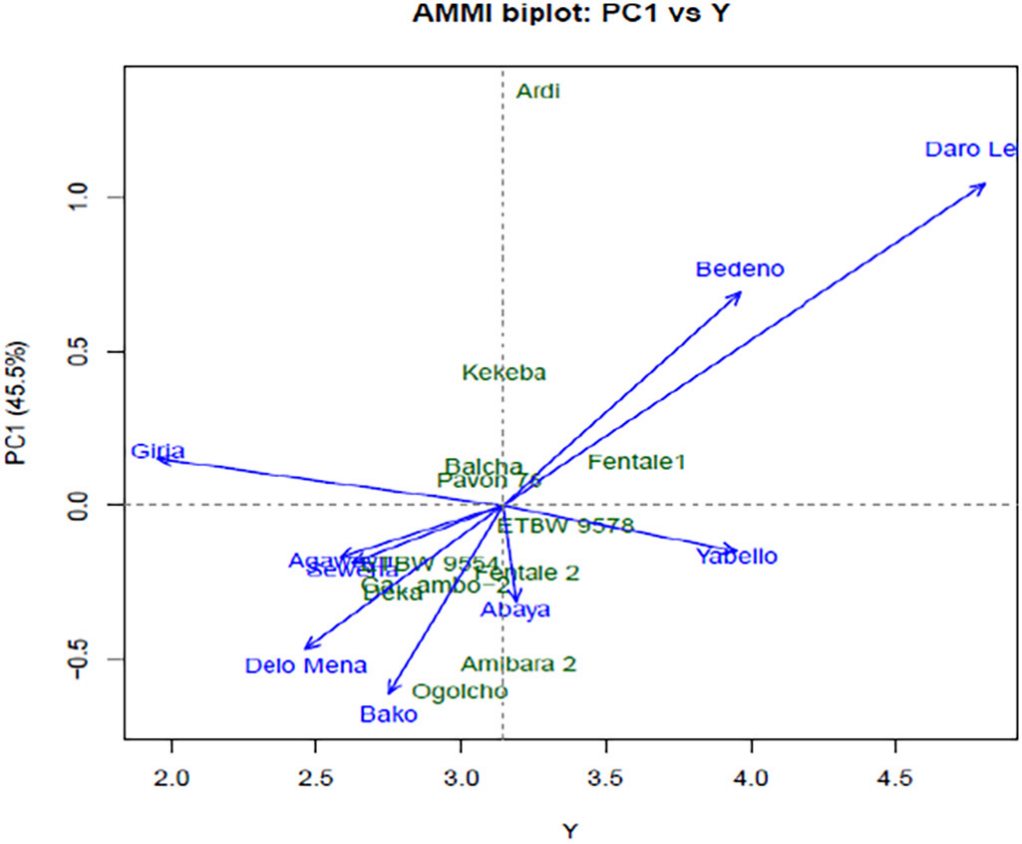
AMMI biplot of interaction principal component axis (IPCA1) against mean grain yield t ha^−1^ (Y) of 12 bread wheat varieties across 11 environments

The first interaction principal component axis (IPCA) and mean grain yield t ha^−1^ were used to construct an AMMI biplot graph to gain sufficient information on the stability of individual genotypes in different test environments (Figure 2). The result of AMMI Biplot analysis with IPCA1 against mean grain yield (t ha^−1^) indicated that most test genotypes showed good stability for grain yield in most test environments. However, Ardi and Ogolcho were the most unstable genotypes. Previous studies showed that, the IPCA scores approximate to zero, the more stable the genotype is all over the test environments (Purchase et al., 2000). The ideal genotype is one with high productivity and IPCA1 values close to zero, whereas the undesirable genotype has low stability associated with low productivity (Gauch & Zobel, 1988). Moreover, in this study test environments Daro Lebu, Bedeno, and Yabello were the most productive environment, while Girja was the least productive environments of irrigated bread wheat for lowlands. In the AMMI1 biplot display, genotypes or environments that fall on a perpendicular and horizontal line of the graph had similar mean yield and similar interaction, respectively. On the other hand, genotypes or environments on the left and right‐hand side of the midpoint line have less and higher yield than the grand mean, respectively. The score and sign of IPCA1 reflect the magnitude of the contribution of both genotypes and environments to G × E, where scores near zero are the characteristic of stability and a higher score (absolute value) designate instability and specific adaptation to a certain environment (Gollob, 1968).

### 3.3 AMMI stability values (ASV)

ASV is the distance from zero in a two‐dimensional scatter diagram of IPCA1 scores against IPCA2 scores. Since the IPCA1 score contributes more to the GE sum of square, it has to be weighted by the proportional difference between IPCA1 and IPCA2 scores to compensate for the relative contribution of IPCA1 and IPCA2 total GE interaction sum squares. According to this stability parameter, a genotype with least ASV score is the most stable. The high interaction of genotypes with environments was also confirmed by high ASV and rank, suggesting unstable yield across environments. In general, the importance of AMMI model is in reduction of noise even if principal components do not cover much of the GE‐SS (Gauch, 1992; Gauch & Zobel, 1996).

The AMMI model IPCA1 and IPCA2 scores of grain yield for each bread wheat varieties and the corresponding AMMI stability value (ASV) are shown in Table 4. Based on this analysis, test varieties Pavon 76, ETBW 9554, Fentale 1, and Fentale 2 were the most stable varieties with AMMI stability values (ASV) of 0.34182, 0.39580, 0.41025, and 0.41133, respectively. Test genotypes with least AMMI stability value (ASV) from the origin are regarded as the most stable. This analysis also confirmed that Ardi, Ogolcho, Amibara 2, and Kekeba were the most unstable genotypes in the present study. Besides, the varieties, Deka, Balcha, and Ga'ambo‐2 were moderately stable with AMMI stability value ranging from 0.52085 for variety Deka to 0.73519 for variety Ga'ambo‐2. The quantitative stability value called AMMI Stability Value (ASV), developed by Purchase et al. (2000) to rank genotypes through the AMMI model was considered to be the most appropriate single method of describing the stability of genotypes (Bose et al., 2014; Bavandpori et al., 2015; Tena et al., 2019).

**TABLE 4:** Mean of 12 varieties, AMMI stability values, genotypic selection index, and coefficient of variation

Genotype	Mean grain Yield (t ha^−1^)	ASV	rASV	rYSI	GSI	IPCA 1	IPCA 2
Fentale 1	3.62	0.4103	3	1	4	−0.2182	−0.0765
ETBW 9578	3.37	0.7457	8	2	10	−0.0652	−0.7359
Ardi	3.28	2.5125	12	3	15	1.3475	0.3456
Fentale 2	3.24	0.4113	4	4	8	0.1433	−0.3148
Amibara 2	3.21	0.9872	10	5	15	−0.516	−0.2575
Kekeba	3.16	0.798	9	6	15	0.4318	0.0292
Pavon 76	3.11	0.3418	1	7	8	0.0851	0.3036
Balcha	3.09	0.7152	6	8	14	0.1295	−0.674
Ogolcho	3.01	1.2989	11	9	20	−0.6105	0.6449
Ga'ambo 2	2.92	0.7352	7	10	17	−0.2546	0.5652
ETBW 9554	2.91	0.3958	2	11	13	−0.1908	0.1802
Deka	2.79	0.5209	5	12	17	−0.282	−0.0099

Abbreviations: ASV, AMMI stability value; rASV, Rank of AMMI stability value; rYSI, Rank of yield index; GSI, Genotypic selection index; CV%, coefficient of variation in percentage.

However, stable genotypes would not predictably provide the best yield performance, and therefore identifying genotypes with high grain yield together with consistent stability across growing environments. Therefore, Genotype Selection Index (GSI) which combines both mean yield and stability in a single index have been introduced to further detect high‐yielding genotypes with stable yield performance, through diverse growing environments (Mohammadi & Amri, 2008). Genotype Selection Index (GSI) in the present study showed that the most stable and high yielding exhibited by variety Fentale 2, Fentale 1, Pavon 76, and ETBW 9578, whereas, Ogolcho, Ga'ambo‐2, and Deka were the least stable and low yielding genotypes.

### 3.4 Evaluation of environments and varieties

**FIGURE 3:**
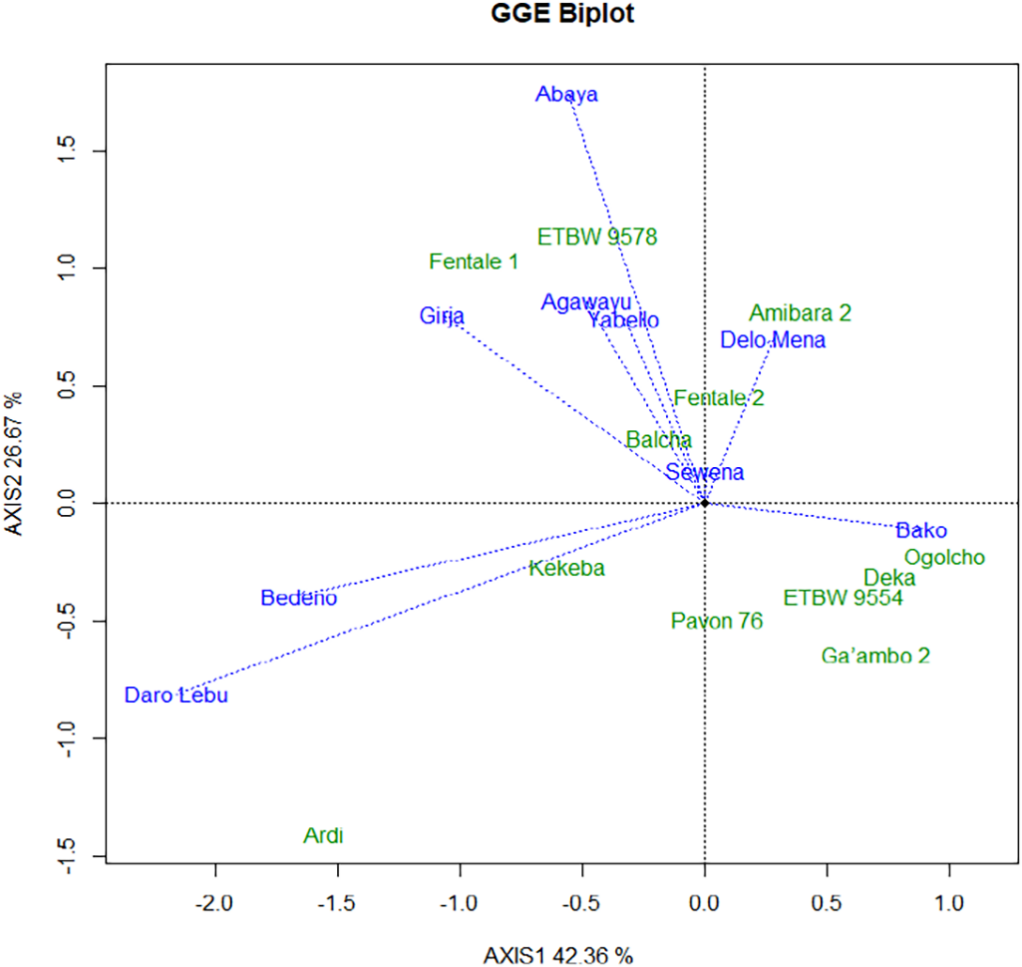
GGE biplot analysis showing the test environments and genotypes

**FIGURE 4:**
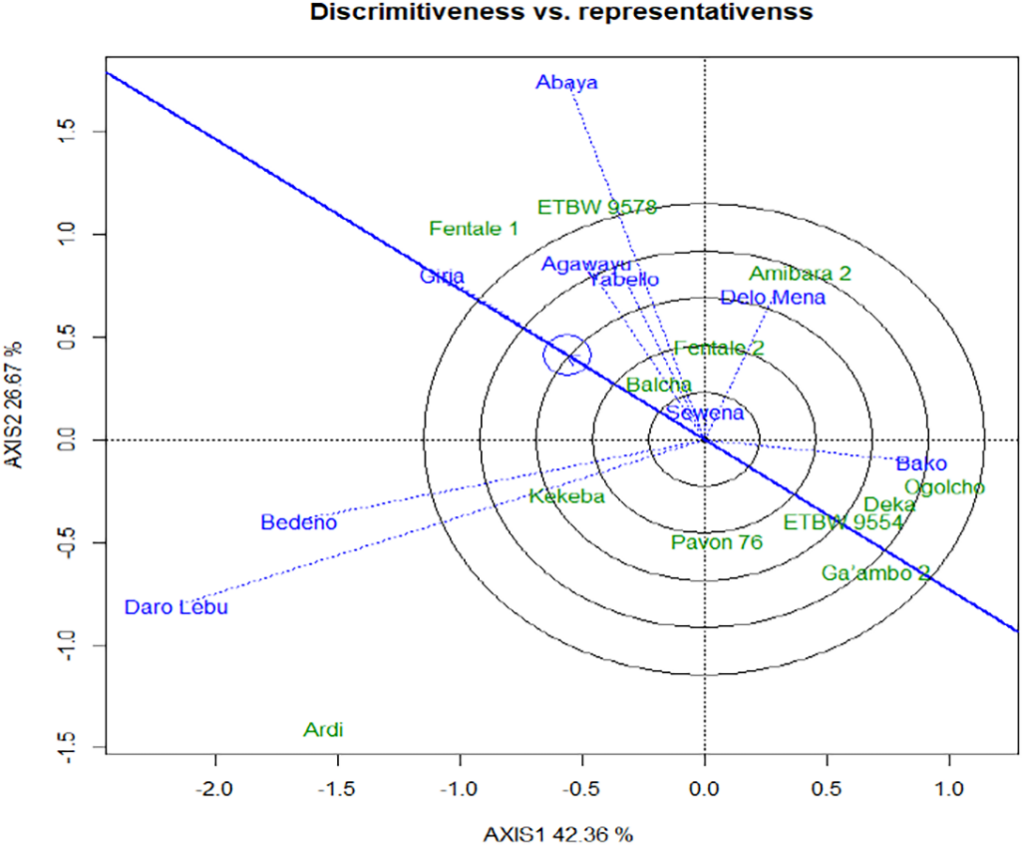
Discrimination and representativeness view of the GGE biplot to show the discriminating ability and representativeness of the test environment

The concentric circles on the GGE biplot help to visualize the length of the environment vectors, which is a measure of the discriminating ability of the environments (Dabessa et al., 2016; Yan & Tinker, 2006). A test environment that has a smaller angle with the Average‐Environment Axis (AEA) is more representative of other test environments (Yan et al., 2011). Nine test environments considered in this study, Daro Labu and Bedeno were the most discriminating, whereas Bako was the least discriminating environment for evaluation of lowland bread wheat for irrigation in the Oromia region (Figures 3 and 4). Evaluation of different genotypes in a multi‐environment is not only important to determine high‐yielding varieties but also to identify sites that best represent the target environment (Yan et al., 2001). Environments (locations) that are both discriminating and representative are good test environments for selecting generally adapted genotypes. The location (Sewena) was the most representative environment, while Daro Labu was the least representative of all test environments.

This study shows that the most discriminating was Girja and representative environment was Sewena for selecting wide adaptable for irrigated wheat varieties. Discriminating test environments are useful for selecting specifically adapted genotypes if the target environments can be divided into different environments (Yan & Tinker, 2006). Test environments that are consistently non‐discriminating (non‐informative) provide little information on the genotypes, therefore, should not be used as test environments (Yan & Tinker, 2006).

### 3.5 Environmental analysis

**FIGURE 5:**
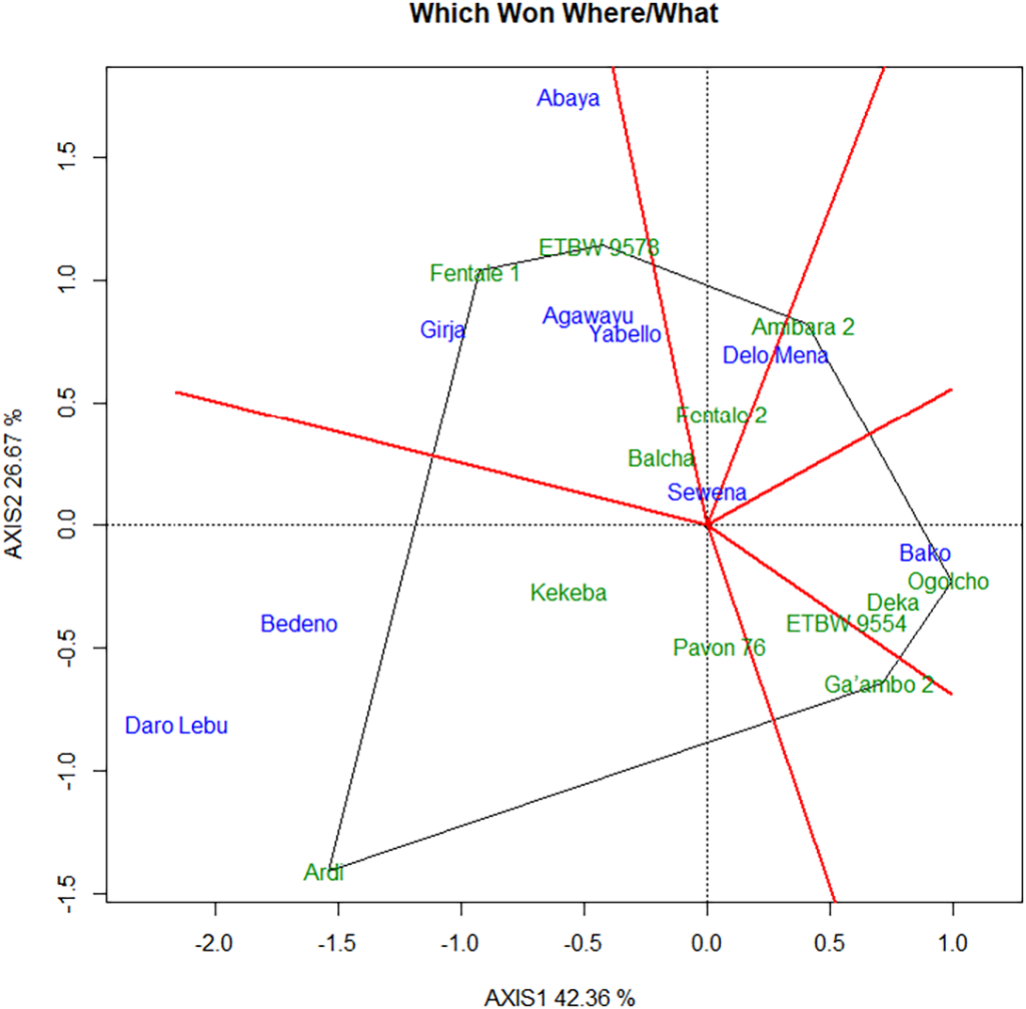
The which‐won‐where view of the GGE biplot to show which genotypes performed best in which environments

The “which‐won‐where” view of the GGE biplot, which consisted of an irregular polygon formed by connecting vertex genotypes and a set of lines drawn from the biplot origin and intersecting the sides of the polygon at right angles, was indicated in Figure 5. The vertex genotypes in this case were Ardi, Fentale 1, ETBW 9573, Amibara‐2, Ogolcho, and Ga 'ambo 2. Figure 5 helps to seek opportunities to subdivide the target environment into sub‐regions (environments). Thus, it classified the environment markers into four sectors (i.e., four environments). This revealed that no single genotype had the highest yield in all environments. Four environments including Yabello, Abaya, Agawayu, and Girja were grouped into the same environment. Daro Lebu and Bedeno were grouped into second environment (sub‐region). The third environment consists Bako; whereas, Sewena and Delo Mena were grouped into fourth environment. The genotype on the vertex of the polygon, contained in a sub‐region, had the highest yield in at least one environment and was one of the best‐performing genotypes in the other environments (Yan & Rajcan, 2002). All other genotypes are contained within the polygon and have smaller vectors, and they are less responsive in relation to the interaction with the environments within that sector. On the other hand, environment IPC1 scores had all positive values leading to non‐cross‐over type G × E interaction. Unlike environment IPC1, environment IPC2 scores had both negative and positive values. This indicated that there was a difference in ranking orders among genotypic yield performances across environments leading to crossover G × E interaction (Figure 3). The same result was consistent with previous reports (Yan, 2011; Tamene & Taddese, 2014).

The distances from the origin (0, 0) are indicative of the amount of interaction exhibited by genotypes over environments or environments over genotypes (Voltas et al., 2002). Unlike the vertex genotypes, those that were located near the biplot origin, variety Balcha, had demonstrated less responsive to the changing environments. Vertex genotypes, those are farthest from the origin, they are either best or poorest in some or all test environments (Yan & Kang, 2003). Therefore, they positively or negatively expressed a highly interactive behavior and contributed more to the exhibited G × E interaction. Thus, vertex varieties Balcha, Fentale 2, and Kekeba were found the best performer however, Ardi, Fentale 1, and ETBW 9578 were the poorest across environments and manifested their high contribution to the existed G × E interaction. Likewise, those near origin environments, for instance Sawena, exhibited nearly additive behavior over genotypic performance (Figure 5). This showed that variety yield in Sawena was highly associated with over all environments mean yield, that is, this environment has average response to all genotypes. In contrast, Abaya, Daro lebu, and Bedeno, with their distance from the biplot origin, showed higher variation. This showed that performance consistency of the varieties over seasons was better at Sawena than at Abaya, Daro labu, and Bedeno. Environments within the same sector of the polygon are assumed to share the same winner genotypes. Accordingly, varieties Fentale 1 and ETBW 9578 were winner in sub‐region Abaya, Agawaya, Girja, and Yabello, whereas, Ardi was a winner genotype in Daro lebu and Bedeno.

## 4 CONCLUSION

Evaluation of lowland bread wheat varieties in a multi‐environment of irrigated area is not only important to determine high‐yielding varieties, but also very essenti to identify the best test sites that represent the target environment. In this study, AMMI stability values (ASV) and genotype selection index (GSI) revealed that the most stable and high‐yielding varieties were Fentale 2 and Fentale 1. AMMI and GGE biplot analyses also indicated that the most discriminating area was Girja and representative environment was Sewena for selecting wide adaptable for irrigated lowland bread wheat varieties. Besides, GGE biplot was also reduced in the nine test environments into four representative sub‐regions for evaluating irrigated lowland bread wheat for wide adaptability in potential irrigated areas. The results of the present study indicated that Fentale 2 and Fentale 1 showed better yield stability across all test environments, therefore, these varieties recommended for the lowlands irrigated areas of the Oromia region.

## CONFLICT OF INTEREST

The authors declare that they have no conflicts of interest.

## Data Availability

Not applicable.
